# Determination of the Bioactive Effect of Custard Apple By-Products by In Vitro Assays

**DOI:** 10.3390/ijms23169238

**Published:** 2022-08-17

**Authors:** Alejandro Rojas-García, Lyanne Rodríguez, María de la Luz Cádiz-Gurrea, Abigail García-Villegas, Eduardo Fuentes, María del Carmen Villegas-Aguilar, Iván Palomo, David Arráez-Román, Antonio Segura-Carretero

**Affiliations:** 1 Department of Analytical Chemistry, University of Granada, 18071 Granada, Spain; 2 Thrombosis Research Center, Medical Technology School, Department of Clinical Biochemistry and Immunohematology, Faculty of Health Sciences, Universidad de Talca, Talca 3460000, Chile

**Keywords:** custard apple by-products, revalorization, phenolic compounds, oxidative stress, enzyme inhibition, platelet aggregation

## Abstract

*Annona cherimola* fruit, known as cherimoya or custard apple, is an exotic fruit from South America but is strongly produced in Andalusia, Spain. Its by-products (seeds and peel) are recognised as important sources of antioxidants, including phenolic acids, flavonoids and procyanidins. Therefore, the aim of this study was to carry out the characterization of its phenolic composition and to in vitro evaluate the bioactivity of custard apple seed and peel. Therefore, high performance liquid chromatography coupled to mass spectrometry (HPLC-ESI-qTOF-MS) was performed in order to tentatively identify their phenolic composition. In the end, 19 compounds were identified and quantified, some of them for the first time in the custard apple matrix. Then, seed and peel total phenolic content, as well as antioxidant properties, radical scavenging capacity (O_2_, NO, HOCl) and inhibition of enzymes involved in different pathologies (hyaluronidase, elastase, collagenase, tyrosinase, acetylcholinesterase and xanthine oxidase), were evaluated. Although both extracts showed almost similar antioxidant capacities, custard apple seed stood out slightly more than peel (171 ± 2 vs. 130.0 ± 0.4 μmol TE/g DE, resp.), especially as ·NO scavenger (IC_50_ 1.5 ± 0.2 vs. 11.8 ± 0.3 mg/L, resp.) and hyaluronidase inhibitor (IC_50_ 170 ± 10 vs. 460 ± 20mg/L, resp.). Finally, the application of extracts on a real human model of platelet aggregation was performed, reporting antiaggregatory effects in agonist-promoted platelet thrombus formation. All these results show that custard apple by-products are stated as interesting sources of bioactive compounds with multiple industrial applications for the development of high-added-value products, such as functional foods, nutraceuticals and cosmeceuticals, promoting the circular bioeconomy of these by-products.

## 1. Introduction

The consumption of fruits and other vegetables has been deeply related to the prevention of several health problems over the years [1,2]. This correlation is due to the presence of phytochemicals, mainly phenolic compounds, which exert antioxidant properties and help to decrease conditions and diseases associated with oxidative stress, e.g., cancer, neurodegeneration or atherosclerosis, among others [2].

Over the last decade, tropical fruits have gained remarkable importance worldwide, mainly due to their exotic taste and great nutritional value. Thus, the determination of their composition and biological activity could be a suitable option to discover new sources of bioactives and thus develop high-added-value products [1].

The custard apple, *Annona cherimola* Mill., is a tropical fruit belonging to the Annonaceae family with an exceptional flavor and traditional uses in ancient medicine. Due to cultivation requirements, its production is not much extended worldwide. Spain is one of the most productive countries [3]. The pulp of this nutritive fruit is known to be rich in vitamins, amino acids, minerals, polysaccharides and phenolic compounds, such as flavanols and procyanidins. However, recent works highlight custard apple by-products, such as seed, peel and leaves as potential sources of flavonoids, phenolic acids and phytosterols, among others [4]. Thanks to this phenolic wealth, these by-products are able to exert significant antioxidant activity, so *A. cherimola* may be the perfect choice for the avoidance of oxidative stress and other related effects such as DNA damage, cell oxidant-induced proliferation, lipid peroxidation, modification of endogenous redox imbalance, etc. [2,5]. Custard apple by-products have been demonstrated to exert a host of beneficial properties: antidiabetic, antitumoral, anxiolytic, antineoplastic, antibacterial, insecticide, storage stabiliser, antiparasitic, antimicrobial, cytotoxic and antidepressant activities, along with others [1,3,4]. Therefore, these by-products could be used for the development of new products with nutritional, pharmaceutical and industrial interests, while reducing the amount of waste generated and reducing the negative impact on the environment.

Hence, the goal of the present study is to determine the therapeutic potential of custard apple seeds and peel grown in Andalusia (Spain), since more of the literature has focused on custard apple leaf extract. For this purpose, a comprehensive identification of the phenolic composition through HPLC-ESI-qTOF-MS was carried out. Then, different in vitro spectrometric assays were performed to fully evaluate their bioactive profiles. First of all, the quantitative determination of its phenolic content using the Folin–Ciocalteau method was evaluated. Then, their antioxidant activity was assessed using different procedures through different mechanisms, i.e., hydrogen atom transfer (HAT) and single electron transfer (SET) reactions. For this purpose, the ferric reducing antioxidant power (FRAP) and Trolox equivalent antioxidant capacity (TEAC) as SET reactions, and the oxygen radical absorbance capacity (ORAC) as HAT reaction, were carried out. In addition, the antioxidant capacity was also evaluated through radical oxygen species (ROS) and free radical scavenging ability analysis, an indicator of the antioxidant functionality of food [2]. Finally, their inhibitory capacity was tested against different enzymes involved in physiological processes and cell signalling pathways that, under ROS influence, may end up in detrimental phenomena, such as aging, neurodegeneration or heart diseases.

On the other hand, cardiovascular problems, which are considered a leading cause of mortality among adults, are strongly influenced by platelet activation and aggregation. Consequently, interest in antiplatelet drugs from natural sources is gradually increasing, and this activity has been associated with the high content of phenolic compounds. For this purpose, the antiaggregatory effect on platelets of custard apple by-product extracts was also evaluated in this study in order to determine their potential [6].

To the best of our knowledge, the present study is the first that gathers this volume of assays to evaluate the therapeutic potential of custard apple by-product extracts in comparison to the reviewed literature, which highlights the scarce knowledge about *A. cherimola* properties. Therefore, thanks to this report, custard apple seeds and peel by-products are pointed out as potential sources of bioactive compounds for their application in food, pharmaceutic and cosmetic industries.

## 2. Results & Discussions

### 2.1. Characterization of Custard Apple Seed and Peel Extracts by HPLC-ESI-qTOF-MS

**Figure 1:**
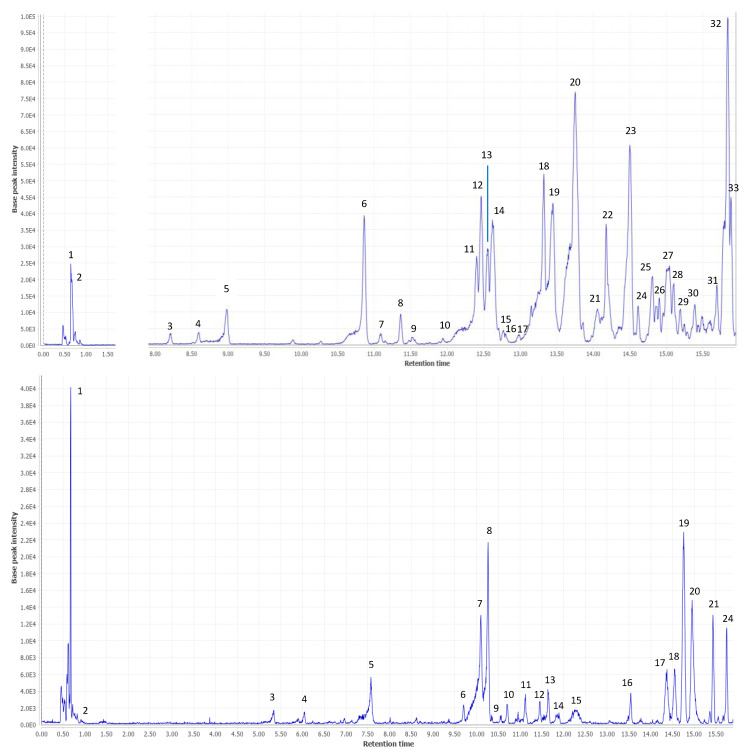
Base peak chromatogram from custard apple seeds (**Top**) and peels (**Bottom**).

After the performance of HPLC-ESI-qTOF-MS, the base peak chromatogram (BPC) is shown in Figure 1 top, bottom. Fifty-five compounds were tentatively identified, some of them for the first time, in both custard apple by-product extracts.

This non-targeted identification was performed based on retention times, mass spectra, fragments, predictions from different software and other studies previously reported in the literature. Of those 55 compounds, seven remained unknown due to the scarce information about them. All the proposed compounds were numbered in order of elucidation and their retention time, together with *m*/*z*, molecular formula, name and, where appropriate, quantification values expressed as mean ± standard deviation in mg of analyte per gram of dry extract (DE). All this information is provided in Table 1.

Custard apple seeds and peel extracts showed diverse compounds such as organic acids, sugars, terpenoids, phenylpropanoids, flavonoids and lignans. The majority of compounds have been identified previously: glycosylated flavonoids such as poncirin (flavanone), miconioside A (flavanone), calabricoside A (flavan-3-ol), kaempferol rutinoside or luteolin glucuronide; rutin; organic quinic and citric acids; hydroxycinnamic acids such as N-caffeoyltyramine and N-feruloyltyramine; peptidic by-products such as cherimolacyclopeptide A and glaucacyclopeptide B; and other compounds, such as sucrose or the triterpenoid kaurenoic acid derivatives [1,3,4]. Nonetheless, some substances found in seed and/or peel composition have never been reported before in the custard apple species itself. From this group, some has been identified in another species from the Annonaceae family, like the glycosidic derivative cleistrioside 5, found in *Cleistopholis patens*, or some lignan derivatives [7]. Others, have only been found in different vegetal compositions, such as litsaglutinan A, a phytohormone derived from abscisic acid, involved in the regulation of seed development [8,9]; different glycosylated flavonoids, such as isomucronulatol [10]; several glycosidic phenylethanoids, such as osmanthuside B and (iso)verbascoside, and the phenylpropanoid magnolenin C, among others [11,12,13,14,15,16,17].

From this tentative characterization, it could be thought that custard apple seed will stand out more than peel due to the higher presence of glycosylated flavonoids and different bioactive molecules and the significant difference between total phenolic contents (272 vs. 22 mg/g DE, respectively). To demonstrate this statement, different in vitro assays were performed.

### 2.2. Evaluation of Total Phenol Content & Antioxidant Capacity Using TEAC, FRAP and ORAC

Phenolic compounds in plants are essential for the development of antioxidant effects by virtue of their ability of chelation, inactivation and prevention on different cell signalling and gene expression pathways [18]. This biological capacity is determined by different parameters, the chemical structure being the most important one. Thus, the presence of different functional groups in determined positions could be crucial for exerting more or less antioxidant activity. In this sense, the quantification, identification and bioactivity evaluation of custard apple by-products would allow the comprehensive study of their possible health benefits.

Therefore, a host of in vitro technologies were carried out. Folin–Ciocalteu enables a semi-quantitative approximation of the phenolic richness of the extract. TEAC and FRAP are both based on a single-electron transfer mechanism, while ORAC is based on hydrogen atom transfer ability. Although all three evaluate antioxidant activity, they act from different points of view, allowing a more complete understanding of the antioxidant capacity of custard apple extracts.

Table 2 shows the phenolic content of each by-product. According to the results, although barely any differences were shown between an *A. cherimola* seed and peel (30.4 and 28.771 mg GAE/g DE, respectively), the seed showed a better TPC value. In concordance, slight differences are demonstrated between by-products for antioxidant capacity, especially for FRAP and ORAC assays. On the contrary, TEAC results indeed showed more contrast between seed and peel, with the former standing out. Such increased TPC and antioxidant results for the seed may have occurred as a consequence of a higher presence of polyphenols than in custard apple peel, e.g., more glycosylated flavonoids and other types of bioactives. A direct correlation between phenolic content and antioxidant activity has been previously demonstrated in other vegetable matrixes [19].

The TPC, FRAP, TEAC and ORAC values in the peel and seeds of the “Fino de Jete” *A. cherimola* variety have been scarcely reported in the literature. Dilrukshi et al. (2020) also determined TPC and the total antioxidant capacity through a FRAP assay on custard apple seed extract and concluded a TPC value of 3.79 mg GAE/g DE and a FRAP value of 0.018 mmol TE/g DE [20]. From the differences observed, it can be determined that our tests yielded more promising results, a trend that was also confirmed by reviewing other works [21,22]. Nevertheless, hardly any literature has been found about the antioxidant properties of custard apple by-products. The contrast observed among antioxidant activities must be associated with different factors that affect the recovery of bioactive compounds. Sample nature, preconcentration technologies, extraction systems and parameters stablished, assays methodologies, and sample origin and supply source are issues that need to be taken into account [23,24].

By comparison, of all the assays performed under equal conditions, custard apple seed extract showed remarkable antioxidant activities, which could be explained by the higher concentration in gallic acid equivalents from the Folin–Ciocalteau method. Nevertheless, other studies that we reviewed have concluded exactly the opposite: custard apple peel extract is richer in phenolic and flavonoid content, with a higher presence of flavan-3-ols and procyanidins in its matrix [1,25]. As aforementioned, differences in the assay execution, as well as the selection of the fruit variety, can bring these inequalities.

Taking into account that TPC, FRAP and ORAC values from seed and peel were almost indistinguishable, in addition to the fact that both possess notable phenolic richness, both extracts are highlighted as interesting raw materials for developing in the food and pharmacological industries.

### 2.3. Evaluation of Free Radical and ROS/RNS Scavenging Potential

Consequently, in order to fully understand the antioxidant properties of custard apple seed and peel extracts, an anti-radical activity evaluation was performed, using some typical endogenous reactive oxygen and nitrogen species (ROS and RNS).

Physiologically, low levels of ROS/RNS are required for correct function and maintenance of health. However, high doses promote oxidative stress, which provokes metabolic malfunctions and biological damage [26]. Species such as superoxide radical (·O_2_^−^), nitric oxide radical (·NO) or hypochlorous acid (HOCl) are produced during regular physiologic processes such as the respiratory chain or inflammation, and all of them lead to potentially damaging host tissue [27,28,29].

Therefore, in order to completely determine the antioxidant profile of custard apple extracts, their free radical scavenging capacity is tested. Table 2 shows the quantity of custard apple by-products needed to inhibit half the concentration of reactive species (IC_50_). Regardless of superoxide radical inhibition evaluation, where no data could be obtained, the seed showed great anti-radical and scavenging ability. Poncirin, a natural flavonoid, has been demonstrated to reduce oxidative damage, diminishing the effect of different radicals and reactive species [30]. This could explain the seed lower IC_50_ values for ·NO and HOCl, which are not far from standard control gallic acid (GA) and epicatechin (EPI) results, gathered in Table 3. In particular, NO inhibition by GA and custard apple seed is closely similar (1.4 vs. 1.5 mg/L, respectively), which exposes the natural extract as a great option for this therapeutic target.

Up to date, there is scarce literature about the anti-radical activity of custard apple seed and peel extracts, demonstrating that deep investigation is needed in this field. ·O_2_^−^ scavenging was impossible to assess since neither the custard apple seed nor the peel showed anti-radical activity against the superoxide specie, which is in agreement with another study formerly performed [31]. Here, a direct relationship is established between ·O_2_^−^ scavenging capacity and flavonoid content, so it can be thought that their presence in the custard apple matrix is not high enough to scavenge this species. However, remarkable results were obtained from nitric radical and hypochlorous acid inhibition, especially in custard apple seed. Specifically, the concentration needed to decrease 50% the amount of ·NO is less than 2 mg per litre, showing high scavenging capacity, never reported before. Concerning other fruits from the Annonaceae family, further anti-radical studies have been carried out on custard apples. *A. squamosa, reticulata* or *muricata* are the main tested species, especially their pulp and leaves, so no information was found about their seeds or peel either. Leaf extract from these fruits did show inhibitory activity against O_2_^−^, and also against ·NO, but quite lower than custard apple extracts [32,33,34].

### 2.4. Evaluation of Enzymatic Inhibition Capacity

Aging is a complex physiological process as a consequence of endogenous and exogenous factors. Intrinsically, oxidative reactions are crucial for cells to function; yet, when their overproduction is exceeded, cells are led to apoptosis and oxidative stress is caused in the organism [35]. This condition unavoidably conducts the overactivation of different enzymes, which end up causing injurious actions on the human body.

In skin, this enzyme imbalance leads to the degradation of extracellular matrix (ECM) and the depletion of different important compounds such as elastin fibers, hyaluronic acid and collagen [36,37]. Decreased concentrations of these compounds affect skin integrity and induce wrinkled skin. Moreover, an abnormal accumulation of melanin, the major human defensive system against UV light, can result in disorders related to hyperpigmentation (melasma, freckles, actinic damage, etc.). Tyrosinase is the key enzyme in melanin biosynthesis, so its malfunction is closely related to this darkening of the skin [37,38].

In the brain and nervous system, oxidative stress has also been considered as one of the main causes of neurodegeneration [35]. One of the major neurodegenerative mechanisms is the overstimulation of acetylcholinesterase (AChE), which breaks down far more of the acetylcholine needed for normal brain function [29]. One of the main causes of oxidative stress in the brain, and in organisms in general, is xanthine oxidase (XOD), since it plays an important role in different metabolism pathways [39]. Many diseases have been associated with XOD activity (hyperuricemia, diabetes, hypertension, ischemia, cardiovascular diseases, etc.), so it is considered an interesting target for the synergic treatment of several pathologies [40].

So, the downregulation of enzymes such as elastase, hyaluronidase, collagenase, tyrosinase, XOD and AChE is postulated as a great defensive strategy against the damaging effects of free radicals. In this sense, different studies have previously established a relationship between phenolic composition and bioactivity of plant-derived extracts in terms of inhibition capacity [29,41]. Table 2 shows results obtained from the evaluation of the blocking capacity of the aforementioned enzymes, expressed majorly as an IC_50_ value. The most remarkable inhibitory effect of both seed and peel extracts was seen against XOD, with IC_50_ values lower than 10 mg/L.

After the enzyme-inhibition assessment, it could be appreciated how functional extracts are against XOD activity, especially the peel one. Other less remarkable results were reported, such as both extracts against tyrosinase and the seed extract against hyaluronidase. Generally, the inhibitory power shown by seeds and peels was far below the minimum required to be considered as biologically active extracts, at least under the reaction conditions utilized in those studies, except for XOD. It has been reported previously that the presence of a double bond between C2 and C3, and free hydroxy groups on C5 and C7 in flavonoid skeletons promotes high xanthine oxidase inhibition activity, which is consistent with the presence of rutin and quercetin compounds in the peel matrix [42]. In the case of AChE, again, poncirin seems to explain differences among seeds and peels since its structural characteristics (4′-methoxyl group and 7-O-sugar moiety) are considered essential for AChE inhibition [43]. Moreover, Wang et al. (2022) also reported anti-neurological damage caused by poncirin flavonoid trough anti-inflammation mechanisms, inhibiting NOX4-mediated NLRP3 inflammasome activation [30]. This could highlight custard apple seeds as a remarkable source of different neuroprotective compounds.

The comparison of these results with previous assays was impossible since hardly any literature could be found about this issue. Only Galarce-Bustos et al. (2020) studied the ability to inhibit AChE of “Fino de Jete” custard apple peel extracts, reporting an IC_50_ value of near to 288 mg/L [22]. In this study, it was impossible to reach measurements at such high concentrations, probably due to interferences caused by the colour intensity of samples. More evaluations have been carried out on other species from the Annonaceae family. For instance, Chatatikun et al. (2017) examined the anti-tyrosinase and anti-collagenase activities of *A. squamosa* leaf extract at 1000 mg/L, showing 22 and 55% inhibition, respectively [36]. Hendriani et al. (2016) also assessed XOD inhibition by *A. muricata* leaf extract, with an IC_50_ of 102 mg/L [44]. In both cases, the custard apple seeds and peel extracts studied here showed lower IC_50_ and, hence, more potent inhibitory activity against the enzymes tyrosinase and collagenase than leaf extracts tested before.

Finally, compared to the standard controls shown in Table 3, XOD inhibition was performed better by custard apple extracts than by EPI, confirming their enzymatic potential. In addition, seed extract performed higher enzymatic inhibition against hyaluronidase than controls GA and EPI and, in case of tyrosinase, custard apple inhibitory capacity was lower than the control kojic acid, but relatively close. 

### 2.5. Evaluation of Platelet Antiaggregatory Activity

The antiplatelet activity of custard apple seeds and peel extracts (1 mg/mL) was evaluated by turbidimetry. Platelet aggregation was stimulated with ADP (4 µM), TRAP-6 (10 µM) and collagen (1 µg/mL), as can be seen in Table 4. These agonists act on different platelet receptors. It was observed that custard apple peel and seed extracts significantly inhibited platelet aggregation stimulated by the studied agonists, although this effect was greater compared to TRAP-6 and collagen for the two study extracts. When platelet aggregation was stimulated with ADP, both extracts had lower antiplatelet potential, 26 ± 1% in the case of custard apple peel and 34 ± 1% in the case of custard apple seeds. Except for this agonist, both extracts are highlighted in comparison to positive control adenosin, especially in TRAP-6 stimulation.

**Figure 2:**
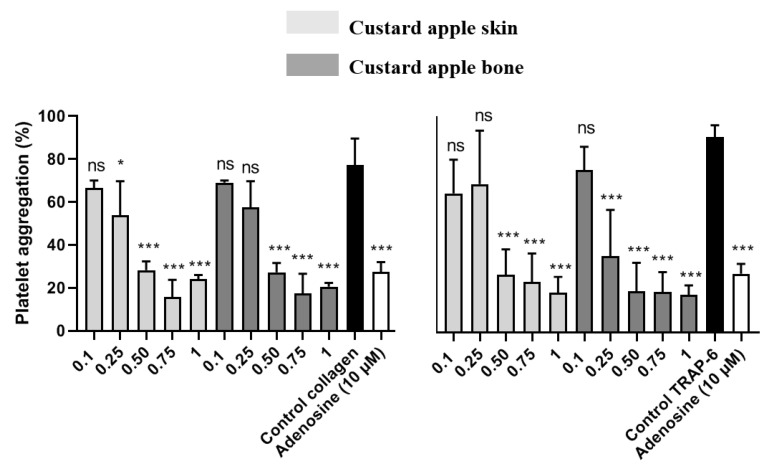
Study of platelet aggregation of custard apple skin (peel) and bone (seed) extracts induced by collagen and ADP. The PRP was previously incubated with vehicle or avocado extract (0.1, 0.25, 0.50, 0.75 and 1 mg/mL). After 3 min of incubation at 37 °C, it was stimulated with the agonist to initiate platelet aggregation for 6 min. The negative control is in the absence of the extracts. Bar graph indicates maximum aggregation expressed as a percentage (mean ± SEM; *n* = 6). Differences between groups are analysed by ANOVA using Dunnet’s post-hoc test. ***: *p* < 0.001 and *: *p* < 0.01 denote statistically significant differences compared to the vehicle; ns: non-statistical difference with respect to the vehicle (PBS).

Lower concentrations of the extracts were also evaluated to study the concentration-dependent antiplatelet effect. Thus, the extracts that showed the highest percentage of platelet aggregation inhibition, greater than 50%, were selected to evaluate the antiplatelet activity. Figure 2 shows the antiplatelet activity of custard apple peel and seed extracts stimulated by TRAP-6 and collagen.

As can be seen, custard apple peel extract significantly inhibited TRAP-6-stimulated platelet aggregation compared to the control up to a concentration of 0.50 mg/mL, while the antiplatelet potential against collagen was higher, obtaining a significant inhibition up to 0.25 mg/mL. On the other hand, custard apple seed extract inhibited platelet aggregation against TRAP-6 up to 0.25 mg/mL. Both extracts showed a concentration-dependent antiplatelet potential.

**Figure 3:**
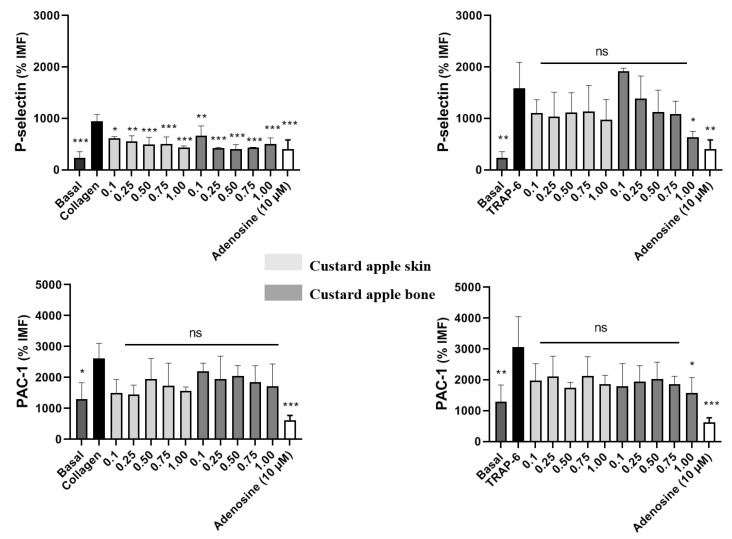
Effect of e of custard apple skin (peel) and bone (seed) extracts on the expression of platelet activation markers: (**Top**) Effect on P-selectin expression and (**Bottom**) Effect on PAC-1 expression. Platelets were stimulated with ADP or Collagen. Platelets were identified as a CD61 + population. Statistical analysis was performed by ANOVA (Dunnet’s test). * *p* < 0.05, ** *p* < 0.01 and *** *p* < 0.001 vs. Vehicle (PBS), vs. activated control (agonist) (*n* = 5).

These studies were conducted to determine the effect of custard apple peel and seed extracts on platelet activation stimulated by the agonists TRAP-6 and collagen, activated condition, 100% expression of *p*-selectin and activation of GPIIb/IIIa (Figure 3). Custard apple peel and seed extracts significantly inhibited collagen-stimulated p-selectin expression up to the lowest concentration evaluated, 0.1 mg/mL. This effect was concentration-dependent. However, when platelets were stimulated with TRAP-6, only custard apple seed extract inhibited p-selectin expression and only at the highest concentration studied, 1 mg/mL. Similarly, only custard apple seed extract inhibited the activation of GP IIb/IIIA induced by TRAP-6.

These results suggest that custard apple peel and seed extracts inhibit platelet activation by reducing P-selectin expression. Nevertheless, only custard apple seed extract was able to decrease the activation of GP IIb/IIIA at the highest concentration studied. Antiplatelet activity has been reported before in other species from Annonaceae family (*Annona squamosa*) mainly due to the presence of ent-kaurene diterpenoids, which have been identified in custard apple peel [45]. This could explain better antiplatelet results shown by this by-product compared to seed.

## 3. Materials and Methods

### 3.1. Chemical Reagents

For extractions and solutions, ultrapure water was obtained with a Milli-Q system Millipore (Bedford, MA, USA) and absolute ethanol was purchased from VWR chemicals (Radnor, PA, USA).

The following reagents were provided from the indicated suppliers: sodium carbonate, acetic acid, TPTZ (2,4,6-tris(2-pyridyl)-s-triazine), sodium hydroxide and hydrochloridic acid were purchased from Fluka (Honeywell, NC, USA). Absolute ethanol and sulfuric acid were purchased from Riedel-de-Haën (Honeywell, NC, USA). Sodium hypochlorite solution EMPLURA was purchased from Merck (Darmstadt, Germany). NOC-5 was purchased from Chemcruz (Santa Cruz Biotech., Dallas, TX, USA). Gallic acid, Folin reagent, ABTS (2,2′-azinobis (3-ethylbenzothiazoline-6-sulphonate)), potassium persulfate, Trolox (6-hydroxy-2,5,7,8-tetramethylchroman-2-carboxylic acid), sodium acetate, ferric chloride, heptahydrate ferrous sulphate, fluorescein, AAPH (2,2′-azobis(2-amidinopropane) dihydrochloride), sodium phosphate monobasic and dibasic, DHR (dihydrorhodamine), DMF (dimethylformamide), potassium dihydrogen phosphate anhydrous, NADH (β-nicotinamide adenine dinucleotide), NBT (nitrotetrazolium blue chloride), PMS (phenazine methosulfate), DAF-2 (diamino fluorescein diacetate) tyrosinase inhibitor screening kit (colorimetric), Tris (tri (hydroxymethyl) aminomethane), acetylthiocholine iodide, 5.5-dithiobis-(2-nitrobenzoic acid), acetylcholinesterase from Electrophorus, Cayman’s xanthine oxidase fluorometric assay kit, neutrophil elastase colorimetric drug discovery kit, sodium chloride, hyaluronic acid, hyaluronidase from sheep testes, tricine, 1-10 phenantroline, collagenase from Clostridium histolyticum and FALGPA (N-[3-(2-furyl)acryloyl]-L-leucyl-glycyl-L-prolyl-L-alanine) were purchased from Sigma-Aldrich (St. Louis, MO, USA).

### 3.2. Extraction of Custard Apple Agro-Industrial By-Products

Fresh custard apples of the “Fino de Jete” fruit variety were kindly donated by the commercial group La Caña, Miguel García Sánchez e Hijos, S.A. (Motril, Spain). In the first place, the by-products seeds and peel from each fruit were completely and manually separated and cleaned under the flow of tap water, also removing as much pulp rests as possible adhered to the peel with a knife.

Subsequently, after storage in the freezer for a certain period of time, total raw cleaned custard apple seeds and peels were weighed and then dried at 80 °C for 9 h in an oven with air convection to avoid the proliferation of microbial agents. Once custard apple by-products were sufficiently dried, the grinding phase started in order to minimise particle size to less than 1 mm and thus promote extraction by increasing active surface.

Finally, the extraction step was carried out through a solid–liquid extraction technique: ten grammes of ground custard apple by-product were weighted in glass containers and, as GRAS solvent, 100 mL of ethanol and water in a proportion of, respectively, 80:20 (v:v) were added. Then, several containers were put into a magnetic stirrer at 170 rpm and 45 °C during 2 h so as to assure a suitable mixture, and all the resultant supernatants were collected, filtered and concentrated in a rotary evaporator. Consecutively, to achieve the maximum evaporation to dryness, samples underwent vaporisation under vacuum in a Speed Vac (Thermo Scientific^®^SC 250 exp). Once finished, both samples were weighed for the extraction yield and stored at −20 °C until further use. Extracts showed a caramel-like appearance and texture with a brownish shade. 

The extraction process was performed following previous reports but using several modifications [46].

### 3.3. HPLC-ESI-qTOF-MS Analysis

Custard apple seed and peel extracts at 5000 mg/L were analysed by using high performance liquid chromatography (ACQUITY UPLC H-Class System; Waters, Milford, MA, USA) coupled to electrospray (ESI) quadrupole time-of-flight mass spectrometry (Synapt G2, Waters Corp., Milford, MA, USA). The separation was performed in a ACQUITY UPLC BEH Shield RP18 Column, 130 Å, 1.7 µm, 2.1 mm × 150 mm at a flow rate of 0.7 mL/min using volume injection of 10 μL [47].

The mobile phases were water acidified with acetic acid 0.5% *v*/*v* (A) and acetonitrile (B). In order to accomplish the most efficient separation possible, the multi-step linear gradient was: 0.0 min [A:B 99/1], 2.33 min [A:B 99/1], 4.37 min [A:B 93/7], 8.11 min [A:B 86/14], 12.19 min [A:B 76/24], 15.99 min [A:B 60/40], 18.31 min [A:B 2/98], 21.03 min [A:B 2/98], 22.39 min [A:B 99/1] and 25.0 [A:B 99/1]. At the end of each analysis, initial conditions were kept so as to equilibrate the system before the subsequent injection. Detection was carried out using negative ionisation mode over a mass range from 50 to 1200 *m*/*z*, setting the detection window to 100 ppm. The spectrum was obtained from two parallel scan functions operated at both low and elevated collision energies (4 eV and 20–60 eV, respectively). All operating parameters set are collected here: source temperature 100 °C; scan duration 0.1 s; resolution 20,000 FWHM; desolvation temperature 500 °C; desolvation gas flow 700 L/h; capillary voltage 2.2 kV; cone voltage 30 V; cone gas flow 50 L/h.

In order to treat the data obtained, MZmine 2.53 open-source software and Sirius 4.4.29 were chosen to process and visualise information. By contrasting it with the literature available for both custard apple samples (and other species belonging to Annonaceae family), compound characterisation was mostly achieved. The literature search for published spectral information was performed by using SciFinder^®^.

### 3.4. Quantification of Individual Phenolic Compounds by HPLC-ESI-qTOF-MS

The quantification of identified phenolic compounds was performed using significantly linear (R^2^ > 0.99) calibration curves of respective reference compounds. Then, the standard concentration was plotted as a function of the peak area obtained from the HPLC-ESI-qTOF-MS analyses. For this purpose, a pattern mix was prepared from stock solutions (500 mg/L) of different standards diluted to concentrations of 0.5–500 mg/L. The selected standards were quercetin glucoside, myricetin-3-glucoside, verbascoside, catechin and quinic acid. Most reference compounds were not available in the sample, so the quantification was majorly performed using structurally related substances, considering the phenolic compound standard has a similar aglycon moiety. Quantification values are expressed as mean ± standard deviation in mg of analyte per gramme of dry extract (DE) [47]. Table 5 summarises analytical parameters:

### 3.5. In Vitro Assays for Bioactive Determination of Phenolic Compounds in Custard Apple By-Products

All undermentioned assays performed were adapted to a 96-well polystyrene microplate and the absorbance measurement was carried out on a Synergy H1 Monochromator-Based Multi-Mode microplate reader (Bio-Tek Instruments Inc., Winooski, VT, USA). Moreover, the methodologies used were performed following previous papers [47].

#### 3.5.1. Evaluation of In Vitro Antioxidant Potential

Antioxidant properties of potential by-products were evaluated by the assays FRAP, TEAC and ORAC. In addition, the total phenolic content (TPC) was determined according to the Folin–Ciocalteau method. All these procedures were described previously in different studies; here, they were performed following them [48,49,50,51].

FRAP, TEAC and TPC assays were based on absorbance measurements, using wavelengths of 593,734 and 760 nm, respectively. ORAC, in turn, was based on fluorescence measurement, disposing of 485 and 520 nm as excitation and emission wavelengths, respectively. All measurements were made in triplicate.

#### 3.5.2. Evaluation of Free Radical and ROS Scavenging Potential

As previously reported, while only superoxide was evaluated by the colorimetric method, a fluorometric-based assay was used for nitric oxide and HOCl [47]. The results were expressed as the custard apple by-products extract concentration needed to inhibit the ROS/RNS formation by half (IC_50_). In the case of O_2_^−^, NBT reduction to diformazan is the measured parameter; for NO, oxidation induction of DAF-2, and for HOCl, oxidation induction of DHR to rhodamine [27,52].

#### 3.5.3. Evaluation of Enzymatic Inhibition Potential

All tests were carried out in triplicate, and the IC_50_ was calculated using different custard apple by-products extract concentrations. Procedures were carried out following previous studies [41,53,54,55].

For the evaluation of tyrosinase and xanthine oxidase inhibition potential, full-prepared kits were used. The “Tyrosinase Inhibitor Screening Kit (Colorimetric)” (MAK257, Sigma-Aldrich, USA) is based on the oxidation of tyrosine, producing a chromophore that can be detected at 510 nm and establishing an inhibition control using kojic acid. “Cayman’s Xanthine Oxidase Fluorometric Assay Kit” (Item No. 10010895, Cayman Chem. Co., Ann Arbor, MI, USA) is based on the measure of fluorescence from compound resorufin as of oxidation of hypoxanthine by XO.

### 3.6. Evaluation of Platelet Antiaggregatory Potential

The extracts were lyophilized and dissolved in phosphate buffered saline (PBS) for platelet aggregation studies. Steps were followed as previous papers [47].

#### 3.6.1. Antiplatelet Activity of Custard Apple Seed and Peel Extracts

Increased platelet activity is a descriptor of the risk of cardiovascular events in healthy men and in patients with coronary artery disease. The antiplatelet activity on human platelets of custard apple peel and seed extracts was studied using turbidimetry according to the methodology described by Born et al. [56]. Blood samples were obtained from six healthy volunteers who previously signed informed consent and did not consume antiplatelet non-steroidal anti-inflammatory drugs (NSAIDs) or other medications [57]. Donors signed informed consent according to the protocol approved by the Scientific Ethics Committee of the University of Talca (protocol No. 19/2018), following the Declaration of Helsinki. The blood was centrifuged at 240× *g* for 10 min to obtain Plasma Rich Platelet (PRP), DCS-16 Centrifugal Presvac RV. Then the platelets were adjusted using platelet poor plasma (PPP) to 200 × 109 platelets/L using a hematologic counter (Bayer Advia 60 Hematologic System). PPP was obtained by centrifuging at 800× *g* for 10 min. To measure the antiplatelet effect of samples, a lumi-aggregometer (Chrono-log, Havertown) was used. The adjusted PRP was incubated with PBS (negative control, maximum platelet aggregation) or with custard apple samples at 37 °C for 4 min under constant shaking. Custard apple extracts were first evaluated at 1 mg/mL, then the concentration-dependent antiplatelet effect (0.10, 0.25, 0.50, 0.75 mg/mL) was studied. Platelet aggregation was stimulated with ADP (4 µM), TRAP-6 (10 µM) and collagen (1 µg/mL) for 6 min at 37 °C. RV. Platelet aggregation was measured as the increase in light transmission over 6 min, and results were expressed as percentage aggregation with AGGRO/LINK software (Chrono-Log, Havertown, PA, USA). Inhibition of platelet aggregation was calculated using the following equation [58]:

$$\%\;\mathrm{Inhibition}\;=100-\left(\frac{\%\;\mathrm{Platelet}\;\mathrm{aggregation}\;\mathrm{of}\;\mathrm{samples}}{\%\;\mathrm{Platelet}\;\mathrm{aggregation}\;\mathrm{negative}\;\mathrm{control}}\times 100\right)$$

#### 3.6.2. Study of P-Selectin Expression and Activation of GP IIb/IIIa

The evaluation of platelet activation markers, expression of surface P-selectin (granule membrane protein alpha-140) and quantification of the surface density of glycoprotein (GP) IIb/IIIa may play important roles in the detection of inflammatory responses and thrombotic diseases associated with platelet aggregation [59].

The expression of P-selectin and the activation of GP IIb/IIIa were evaluated by flow cytometry according to the method described by Rodriguez et al. [60] with some modifications. The PRP obtained as described previously was incubated for 6 min with extracts of custard apple peel and seeds at the same concentrations evaluated by turbidimetry (1.00, 0.75, 0.50, 0.25, 0.10 mg/mL). All conditions were activated with ADP (4 µM), TRAP-6 (10 µM) and collagen (1 µg/mL) for 6 min at 37 °C. The non-activated baseline condition (PRP without adding an agonist) was used as a negative control. Then, to evaluate the expression of P-selectin, 30 µL of sample was incubated with anti-CD62PE for 30 min in the dark. To evaluate the expression of GP IIb/IIIa in platelets, the sample was incubated with anti-GP IIb/IIIa PAC-1-FITC antibodies for 30 min. The samples were analysed on an Accuri C6 flow cytometer (BD, Biosciences, Franklin Lakes, NJ, USA). The platelet population was labelled with anti-CD61-FITC and selected for cell size by forward scatter (FSC) versus side scatter (SSC) and CD61 positivity to distinguish them from electronic noise. Measurements were made on platelets from six healthy volunteers.

#### 3.6.3. Statistical Analysis

Anti-platelet aggregation data were analysed using Prism 8.0 software (GraphPad Inc., San Diego, CA, USA) and expressed as mean ± standard error (SEM). Differences between groups were analysed using a one-way analysis of variance (ANOVA) and the Dunnet hoc test.

## 4. Conclusions

After this detailed study, both custard apple seed and peel can be considered as phytochemical compound sources, which would be very useful for their application in the food, pharmaceutical and/or cosmetic industries.

However, seed is the most remarkable by-product as it shows higher phenolic content and higher antioxidant capacity as an electron donor, hydrogen donor or free radical scavenger. It also exerted potent inhibitory activity against enzymes such as XOD or hyaluronidase, as well as antiplatelet activity to prevent thrombus formation, although the peel also stood out in these latter areas. Therefore, depending on the target, different extracts could be used in order to accomplish different benefits. Further studies must focus on exploiting these therapeutical properties from an ecological point of view, without residue generation and improving operational conditions for more efficient revalorization, thus promoting the circular economy of custard apple and its by-products.

## Figures and Tables

**Table 1:** Identification of phytochemical compounds in custard apple seeds and peel extracts with ethanol/water by HPLC-ESI-qTOF-MS.

Peak	RT (min)	[M-H]^−^	Mol. Formula	Main Fragments	Compound	Content (mg/g DE)
Seed						
1	0.64	341	C_12_H_22_O_11_	-	Sucrose	NQ
2	0.69	191	C_7_H_12_O_6_	173, 131	Quinic acid	1.36 ± 0.05
3	8.22	561	C_31_H_46_O_9_	519	Yunnanxane	NQ
4	8.60	529	C_29_H_38_O_9_	487	Taxinine H	NQ
5	8.98	607	C_37_H_52_O_7_	453	Protocatechuoyl alphitolic acid	NQ
6	10.86	298	C_17_H_17_NO_4_	135	Caffeoyltyramine isomer 1	NQ
7	11.09	819	-	-	Unknown 1	NQ
8	11.36	842	-	-	Unknown 2	NQ
9	11.52	605	C_28_H_30_O_15_	283	Methyl-kaempferol-[HMG-(1→3/4)]-hexoside	0.53 ± 0.02
10	11.95	609	C_27_H_30_O_16_	591, 373, 255	Quercetin rutinoside	0.42 ± 0.03
11	12.40	593	C_28_H_34_O_14_	431, 269	Poncirin	27 ± 3
12	12.47	495	C_27_H_34_O_14_	300, 285	Dyhydroxyecdysone	NQ
13	12.55	312	C_18_H_19_NO_4_	148, 190, 290	Feruloyltyramine isomer 1	NQ
14	12.62	591	C_29_H_36_O_13_	445	Osmanthuside B isomer 1	49 ± 3
15	12.75	312	C_18_H_19_NO_4_	148, 190	Feruloyltyramine isomer 2	NQ
16	12.79	625	C_29_H_38_O_15_	301, 165	Isomucronulatol diglucoside	0.48 ± 0.05
17	12.96	595	C_28_H_36_O_14_	591, 445	Magnolenin C	0.8 ± 0.1
18	13.32	893	-	609, 444	Lignan derivative	NQ
19	13.45	607	C_29_H_36_O_14_	444	Miconioside A	32 ± 5
20	13.75	609	C_29_H_38_O_14_	446, 283	Litseaglutinan A isomer 1	NQ
21	14.06	633	-	-	Unknown 3	NQ
22	14.17	623	C_29_H_36_O_15_	461	(Iso)verbascoside isomer 1	5.55 ± 0.02
23	14.50	836	C_38_H_63_N_9_O_10_S	-	Cherimolacyclopeptide A	NQ
24	14.61	641	-	-	Unknown 4	NQ
25	14.81	730	C_35_H_53_N_7_O_8_S	-	Glaucacyclopeptide B	NQ
26	14.91	609	C_29_H_38_O_14_	446, 283	Litseaglutinan A isomer 2	NQ
27	15.04	795	C_40_H_60_N_8_O_9_	-	Peptidic derivative	NQ
28	15.08	298	C_17_H_17_NO_4_	-	Caffeoyltyramine isomer 2	NQ
29	15.17	298	C_17_H_17_NO_4_	-	Caffeoyltyramine isomer 3	NQ
30	15.39	591	C_29_H_36_O_13_	445	Osmanthuside B isomer 2	3.0 ± 0.2
31	15.69	609	C_29_H_38_O_14_	446	Litseaglutinan A isomer 3	NQ
32	15.84	623	C_29_H_36_O_15_	461	(Iso)verbascoside isomer 2	152.3 ± 1.0
33	15.87	958	C_45_H_69_N_9_O_10_S_2_	-	Cherimolacyclopeptide F	NQ
TOTAL 272 ± 8						
**Peel**						
1	0.67	191	C_7_H_12_O_6_	173, 131	Quinic acid	4.4 ± 0.4
2	0.76	191	C_6_H_8_O_7_	-	Citric acid	1.11 ± 0.09
3	5.33	443	C_21_H_32_O_10_	-	Penstemide	NQ
4	6.01	411	C_24_H_28_O_6_	-	Eupomatene B	NQ
5	7.54	395	C_16_H_28_O_11_	-	Nonioside	NQ
6	9.66	741	C_32_H_38_O_18_	300	Calabricoside A	0.48 ± 0.07
7	10.04	607	C_27_H_28_O_16_	300	Quercetin derivative	6.7 ± 0.7
8	10.20	609	C_27_H_30_O_16_	300	Rutin	4.7 ± 0.2
9	10.49	461	C_21_H_18_O_12_	285	Luteolin glucuronide	<LOQ
10	10.65	593	C_27_H_30_O_15_	285	Kaempferol rutinoside isomer 1	0.52 ± 0.02
11	11.12	593	C_27_H_30_O_15_	285	Kaempferol rutinoside isomer 2	0.70 ± 0.06
12	11.46	537	-	-	Unknown 5	NQ
13	11.56	561	C_30_H_26_O_11_	289	Catequin derivative	3.4 ± 0.8
14	11.76	481	C_28_H_34_O_7_	-	Gedunin	NQ
15	12.21	657	C_32_H_50_O_14_	-	Annoglabasin H	NQ
16	13.55	641		607	Hydroxyecdysone glycopyranoside	NQ
17	14.28	751		457	Fargoside A	NQ
18	14.55	957	-	-	Unknown 6	NQ
19	14.68	749	C_36_H_62_O_16_	589	Cleistrioside 5	NQ
20	14.88	680	-	-	Unknown 7	NQ
21	15.35	335	C_20_H_32_O_4_	-	Dihydrokaurenoic acid isomer 1	NQ
22	15.66	335	C_20_H_32_O_4_	-	Dihydrokaurenoic acid isomer 2	NQ
TOTAL 22 ± 4						

RT: Retention Time; NQ: Not Quantified; LOQ: Limit of Quantification.

**Table 2:** Evaluation of total phenolic content, antioxidant capacity and radical scavenging ability of custard apple by-product extracts.

Methodology	CAS Extract	CAP Extract
TPC (mg GAE/g DE)	30.4 ± 0.7	28.771 ± 0.008
FRAP (mmol Fe^2+^/g DE)	0.292 ± 0.005	0.27 ± 0.01
TEAC (μmol TE/g DE)	171 ± 2	130.0 ± 0.4
ORAC (mmol TE/g DE)	0.368 ± 0.005	0.324 ± 0.009
HOCL (mg/L) ^1^	11 ± 2	28 ± 4
O_2_^−^ (mg/L) ^1^	N.A.	N.A.
NO (mg/L) ^1^	1.5 ± 0.2	11.8 ± 0.3
AChE (mg/L) ^2^	26 ± 4	12 ± 1
Tyrosinase (mg/L) ^1^	157.1 *	120 ± 10
XOD (mg/L) ^1^	7.2 ± 0.7	4.4 ± 0.4
Elastase (mg/L) ^3^	800 ± 60	410 ± 30
Hyaluronidase (mg/L) ^1^	170 ± 10	460 ± 20
Collagenase (mg/L) ^1^	660 ± 20	690 ± 30

Data are means ± standard deviation (*n* = 3). ^1^ IC_50_, i.e., quantity (mg/L) of custard apple peel and seed extract needed to decrease by 50% the amount of the reactive species in the assay. ^2^ Percentage of inhibition at 111.11 mg/L (maximum concentration tested). ^3^ IC_25_, i.e., quantity (mg/L) of custard apple peel and seed extract needed to decrease by 25% the amount of the reactive species in the assay. * No standard deviation, only one test was carried out in good terms (*n* = 1).

**Table 3:** Positive controls from radical scavenging and enzymatic inhibitions.

Methodology	GA	EPI	PHY	PHE	ELA	KA
HOCl (mg/L) ^1^	3.8 ± 0.3	0.18 ± 0.01	X	X	X	X
O_2_ (mg/L) ^1^	50 ± 3	70 ± 5	X	X	X	X
NO (mg/L) ^1^	1.4 ± 0.3	0.87 ± 0.02	X	X	X	X
AChE (mg/L) ^2^	X	X	0.043 ± 0.004	X	X	X
Tyrosinase (% inh.) ^3^	X	X	X	X	X	49 ± 6
XOD (mg/L) ^1^	X	9 ± 1	X	X	X	X
Elastase (% inh.) ^4^	X	X	X	X	53 ± 5	X
Hyaluronidase (% inh.) ^5^	<10%	<10%	X	X	X	X
Collagenase (% inh.) ^6^	X	X	X	83 ± 2	X	X

GA: gallic acid; EPI: epicatechin; PHY: physostigmine; PHE: 1, 10-phenanthroline; ELA: elastatinal; KA: Kojic acid; inh.: inhibition. ^1^ Inhibitory Concentration at 50%. ^2^ Inhibitory Concentration at 90%. ^3^ At 21.3 ppm. ^4^ At 51.26 ppm. ^5^ From 6 to 220 ppm. ^6^ At 4500 ppm.

**Table 4:** Study of platelet aggregation induced by ADP, TRAP-6 and collagen.

Extracts	TRAP-6 (10 μM)		ADP (4 μM)		Collagen (1 μg/mL)	
	PA (%)	Inh. (%)	PA (%)	Inh. (%)	PA (%)	Inh. (%)
CAS	21 ± 1 ***	76 ± 1	67 ± 1 **	26 ± 1	24 ± 1 ***	70 ± 2
CAP	15 ± 1 ***	82 ± 1	60 ± 1 ***	34 ± 1	21 ± 1 ***	75 ± 2
Ctrl (−)	88 ± 1	0	94 ± 1	0	82 ± 3	0
Ctrl (+)	27 ± 1	68 ± 1	29 ± 1	68 ± 1	25 ± 1	70 ± 2

Results were expressed as mean ± SEM, *n* = 5. Data were analyzed by one-way ANOVA. Post hoc analyses were performed using Dunnet’s test, ** *p* < 0.01 and *** *p* < 0.001 denote statistically significant differences compared to the negative control (vehicle). ADP: Adenosine diphosphate, Inh.: Inhibition, PA: Percentage of platelet aggregation, SEM: Standard error, TRAP-6: Thrombin-6 receptor activating peptide.

**Table 5:** Quantification data of identified phenolic compounds from custard apple seed and peel.

Standard	LOD (µg/mL)	LOQ (µg/mL)	Calibration Range (mg/L)	Calibration Equations	R^2^
Quinic acid (1)	0.04	0.14	(0.977–7.813)	y = 1099.56 x − 21.48	0.999
Quinic acid (2)	0.04	0.14	(3.906–31.25)	y = 2155.60 x − 5059.59	0.990
Verbascoside (1)	0.09	0.29	(0.488–31.25)	y = 2489.96 x + 688.35	0.996
Verbascoside (2)	0.05	0.15	(31.25–500)	y = 354.82 x + 89225.64	0.997
Catechin	0.46	1.43	(0.977–31.25)	y = 857.50 x − 748.37	0.999
Quercetin glucoside	0.09	0.29	(0.488–31.25)	y = 2820.85 x + 688.34	0.993
Myrecetin-3-glucoside	0.03	0.10	(31.25–250)	y = 19393.44 x + 114909.92	0.993

Limit of detection (LOD) and quantification (LOQ), patterns used to quantify for each compound, linear equations and the coefficient of variation (R^2^).

## Data Availability

Not applicable.
